# Design of galectin-1-conjugated nanoparticles as potential immunomodulatory agents

**DOI:** 10.1039/d5md00539f

**Published:** 2025-09-26

**Authors:** Chandradhish Ghosh, Ling Yao, Marilet Sigler, Santiago Di Lella, Alejandro J. Cagnoni, Gabriel A. Rabinovich, Peter H. Seeberger

**Affiliations:** a Department of Biomolecular Systems, Max Planck Institute of Colloids and Interfaces Am Muehlenberg 1 14476 Potsdam Germany peter.seeberger@mpikg.mpg.de; b Instituto de Química Biológica – Ciencias Exactas y Naturales, CONICET – Facultad de Ciencias Exactas y Naturales, Universidad de Buenos Aires Argentina; c Laboratorio de Glicomedicina, Instituto de Biología y Medicina Experimental (IBYME), Consejo Nacional de Investigaciones Científicas y Técnicas (CONICET) C1428 Ciudad de Buenos Aires Argentina; d Departamento de Química Biológica, Facultad de Ciencias ·Exactas y Naturales, Universidad de Buenos Aires C1428 Ciudad de Buenos Aires Argentina; e CaixaResearch Institute 08022 Barcelona Spain; f Institute of Chemistry and Biochemistry, Freie Universität Berlin Arnimallee 22 14195 Berlin Germany; g Departamento de Química Orgánica, Facultad de Ciencias Exactas y Naturales, Universidad de Buenos Aires C1428 Ciudad de Buenos Aires Argentina

## Abstract

Autoimmune disorders are heterogeneous dynamic conditions characterized by dysregulated immune responses and caused by interruption of tolerogenic circuits. Although immunosuppressive drugs, including biological agents, are effective therapeutic options, several patients do not respond to these treatment or develop resistance mechanisms. Galectins, a family of soluble glycan-binding proteins, play central roles in the modulation of autoimmune inflammation. Galectin-1 (Gal-1), a prototype member of this family, interacts with specific *N*-acetyllactosamine (LacNAc) ligands present in *N*- and *O*-glycans *via* its conserved carbohydrate recognition domain (CRD). The immunomodulatory activity of Gal-1 involves regulation of T cell effector populations, inducing apoptosis of Th1 and Th17 cells, differentiation of tolerogenic dendritic cells and induction of myeloid-derived suppressor cells. To develop a rational galectin-based therapeutic strategy, we evaluated whether Gal-1 retains its function upon multivalent presentation on nanoparticles. Specifically, we report the design strategy, synthesis and characterization of galectin-1-conjugated glucose-stabilized gold nanoparticles, and compare their activities with unconjugated galectin-1. This formulation offers novel opportunities for treating a variety of autoimmune diseases, as well as chronic inflammatory disorders.

## Abstract 2

Galectin-1 conjugated to glucose-stabilized gold nanoparticles retains its immunomodulatory function and provides a promising platform for treating autoimmune and chronic inflammatory diseases.

## Graphical abstract

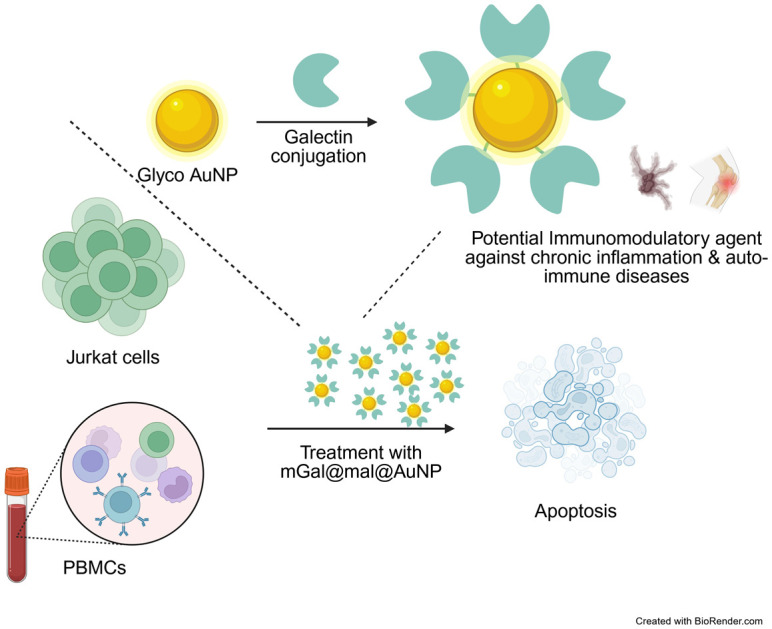

## 1. Introduction

Galectins are a family of soluble lectins defined by a common structural fold and a conserved carbohydrate recognition domain (CRD) with affinity to *N*- and *O*-glycans bearing *N*-acetyllactosamine (Galβ(1–4)-GlcNAc; LacNAc) residues.^1–3^ Galectin-1 (Gal-1), a prototype member of this family, controls numerous immune cell processes including activation, differentiation, trafficking, and survival through specific recognition of LacNAc-bearing glycoconjugates on cell surface glycosylated receptors.^4^ Among these processes, Gal-1 limits the survival of T helper (Th) 1 as well as Th17-differentiated T cells,^5^ favors promotion of tolerogenic dendritic cells (DCs)^6^ and myeloid-derived suppressor cells (MDSCs)^7^ and promotes regulatory T cells.^8,9^ This leads to induction of tumor-immune escape,^6,10,11^ modulation of microbial infections,^12,13^ and resolution of autoimmune inflammation.^14–16^ Structurally, Gal-1 presents only one CRD, a 14.5 kDa subunit (135 aa) that is involved in a homodimerization equilibrium with a reported dimerization constant of 7 μM.^17,18^ Oligomerization of galectins and their multivalent interactions with cell surface glycoconjugates is critical for regulating cellular communication and signalling, by forming ordered galectin-glycan arrays, often termed ‘lattices’, on the cell surface.^19,20^ The linker region structure has also been proven to be critical in this lattice formation.^21^ Particularly, due to the unusually high number of six cysteine residues of Gal-1 in each monomer, this lectin is highly sensitive to oxidative inactivation, which limits its biological activity.^19,20^ Although typically conceived as independent processes, galectin oxidation, activation and oligomerization could be interconnected, favouring multivalent ligand binding.^22^ The key factor appears to be the formation of multivalent galectin-glycan lattices where the bivalent feature of Gal-1 becomes critical. Multivalency is useful to increase the biological function of many proteins, drugs and other biomolecules.^23^ The therapeutic potential of multivalent nanosystems bearing Gal-1 revealed improved function.^24–26^

We have previously developed a simple one-step process that yielded ultra-small glucose stabilized gold nanoparticles, which are highly dispersible in water and can be further functionalized *via* carboxylic acid groups formed by oxidation of sugar residues.^27^ These nanoparticles are efficient drugs carriers for intracellular delivery to treat infections caused by parasites and fungi.^28,29^ Complex carbohydrates, when conjugated to these nanoparticles, exhibit potent immunomodulatory properties without activating the complement system.^30^ Given the excellent properties displayed by these ultra-small nanoparticles, we sought to develop Gal-1-conjugated gold nanoparticles as novel therapeutic agents to ameliorate autoimmune and chronic inflammatory disorders autoimmune disorders. Here, we describe the design and synthesis of Gal-1-conjugated nanoparticles and explore their biological potential.

## 2. Results

### 2.1 Design and synthesis of human Gal-1-bearing nanoparticles

**Fig. 1:**
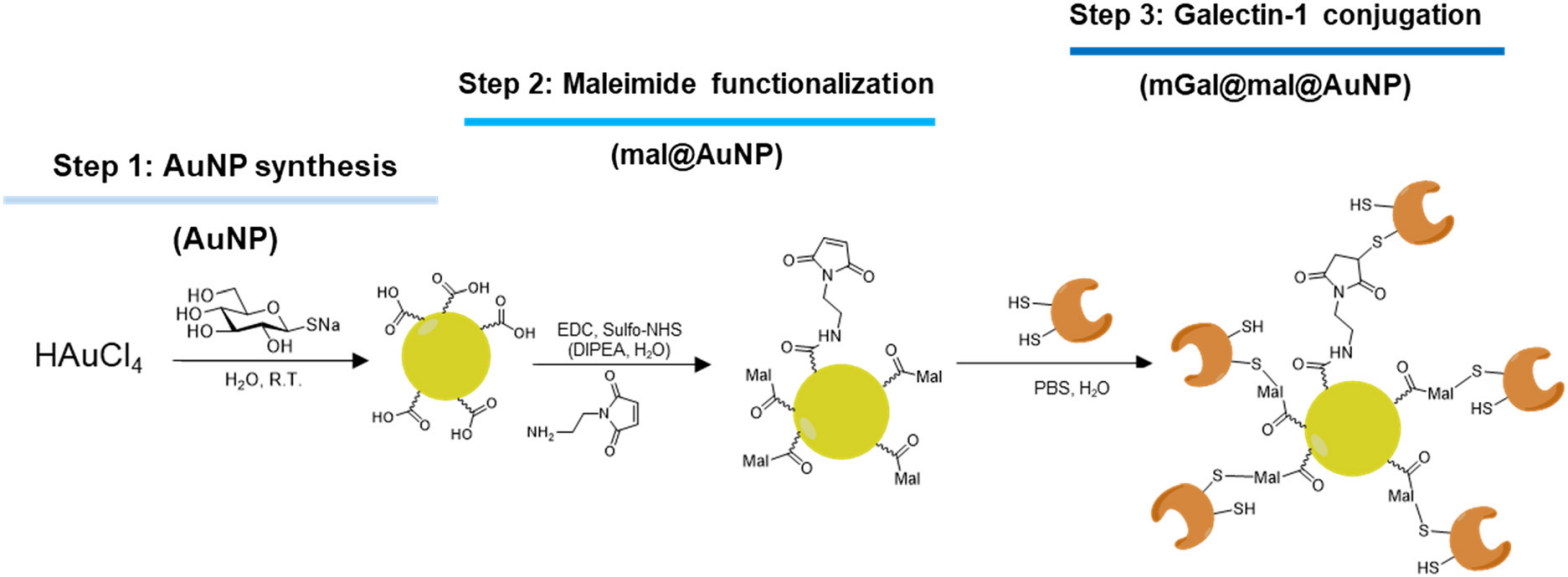
Schematic synthesis of galectin-conjugated nanoparticles. Glucose stabilized gold nanoparticles (AuNP) were first functionalized with terminal maleimide groups using amide coupling chemistry (mal@AuNP). Then the galectin was conjugated to the maleimide functionalized gold nanoparticles using thiol maleimide click chemistry to obtain mGal@mal@AuNP.

In the first step, gold nanoparticles were synthesized by reducing chloroauric acid (HAuCl_4_) in the presence of thioglucose, which served as both the reducing and capping agent (Fig. 1), as previously described.^27^ During the reduction process, thioglucose is partially oxidized, introducing carboxylic acid functionalities on the nanoparticle surface (Fig. 1), which facilitates subsequent chemical modifications.

In the second step, the glucose stabilized gold nanoparticles (AuNP) were functionalized with terminal maleimide to enable covalent protein conjugation. Carboxyl groups on the surface of the glycogold nanoparticles (AuNP) were activated using 1-ethyl-3-(3-dimethylaminopropyl)carbodiimide (EDC) in combination with *N*-hydroxysulfosuccinimide (sulfo-NHS). After activation, a maleimide-functionalized linker was added to the solution and the reaction was subjected to 2 hours of sonication to facilitate efficient coupling. The resulting maleimide-functionalized nanoparticles (mal@AuNP) were purified by overnight dialysis against Milli-Q water and subsequently filtered through a 0.45 μm membrane to remove unreacted materials and aggregates.

Finally, for protein conjugation, recombinant human Gal-1 (hereafter referred to as mGal for consistency with our internal nomenclature) was employed in the third step. The protein was added to the mal@AuNP solution and the reaction mixture was gently stirred overnight at room temperature to allow thiol-maleimide click chemistry to occur, yielding the final protein–nanoparticle conjugates (mGal@mal@AuNP). Conjugates were purified by dialysis to remove unbound protein and residual reagents.

The resulting mGal@mal@AuNPs were characterized by ultraviolet-visible (UV-vis) spectroscopy, infrared (IR) spectroscopy, transmission electron microscopy (TEM), and atomic force microscopy (AFM) to confirm successful functionalization and conjugation.

### 2.2 Characterization of Gal-1-conjugated nanoparticles

**Fig. 2:**
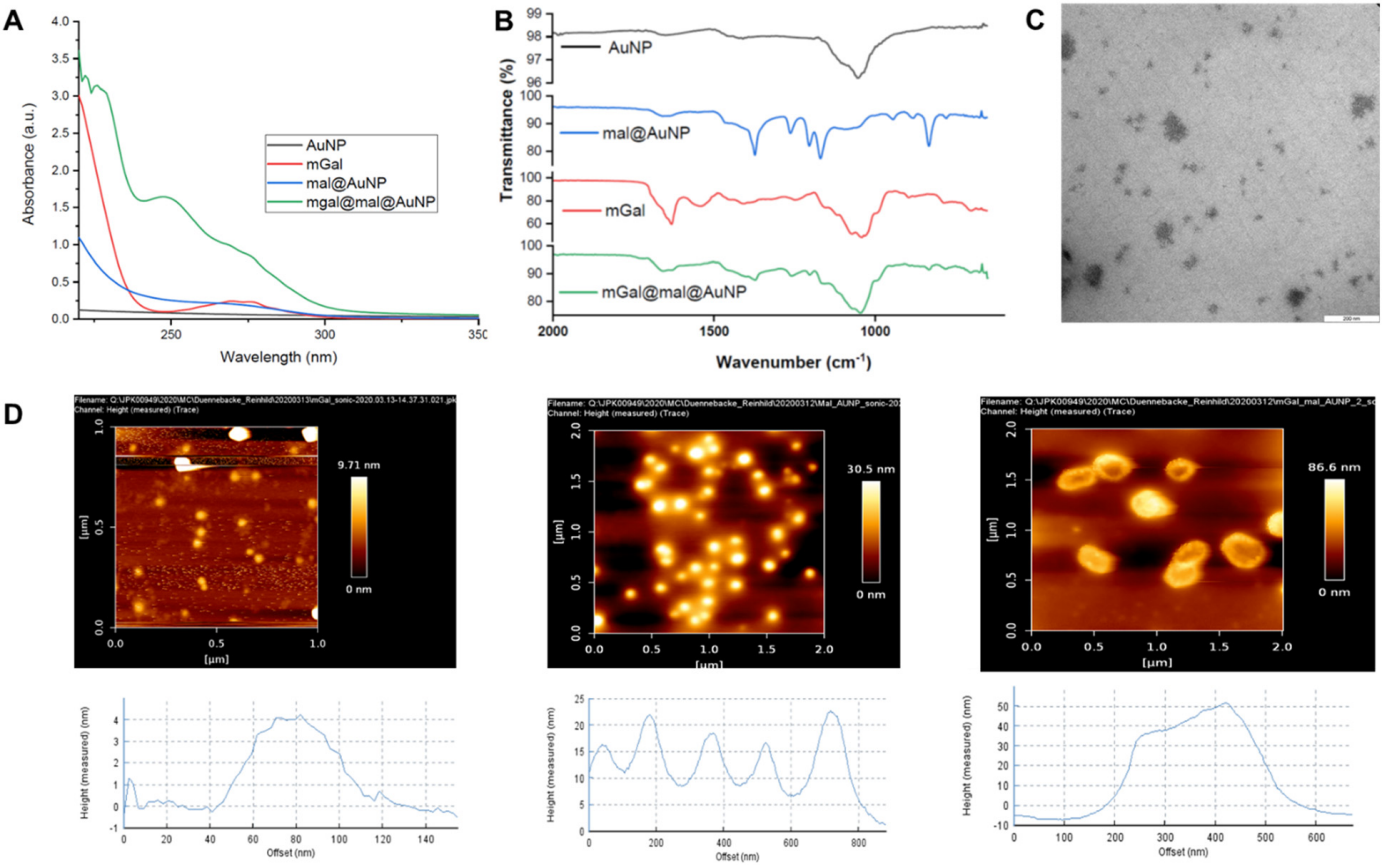
Characterization of galectin conjugated nanoparticles. (A) UV spectra of mGal@mal@AuNP show a 280 nm characteristic signal of protein conjugates. (B) IR spectra of mGal@mal@AuNP show prominent characteristics of both mGal and mal@AuNP. (C) TEM images of mGal@mal@AuNP show presence of 50 nm particles and other small particles. (D) AFM images of naked AuNP, mal@AuNP and mGal@mal@AuNP, respectively. The mGal@mal@AuNP show oval shaped structures of size 50 nm.

Protein–nanoparticle conjugation was confirmed by different spectroscopic and microscopic techniques (Fig. 2). First, ultraviolet (UV) absorption spectroscopy of the unbound protein showed standard peaks at around 280 nm (Fig. 2A). While the naked AuNP (mal@AuNP) showed no absorption in that region, mGal@mal@AuNP absorbed strongly between 250 and 280 nm. In addition, infrared (IR) spectroscopy also confirmed that mGal@mal@AuNP presented the signatures of both mGal and mal@AuNP (Fig. 2B). Next, transmission electron microscopy (TEM) was employed to determine the size of the gold clusters of mGal@mal@AuNP (Fig. 2C). In addition to 50 nm particles, other small particles were observed, possibly a result of radiation damage (Fig. 2C). Moreover, atomic force microscopy (AFM) was used to determine the actual particle size (Fig. 2D). The height of the Gal-1-conjugated nanoparticles from the AFM scan revealed a variation in size from 37–40 nm (Fig. 2D), while that of the unconjugated AuNP and mal@AuNP was around 4 nm and 10–20 nm, respectively. These purified nanoparticles were then studied for their biological activity. For simplicity, we used the protein concentration, as determined from the UV absorption studies as reference for all further studies.

### 2.3 Gal-1-conjugated nanoparticles induce apoptosis of activated peripheral blood mononuclear cells (PBMCs)

**Fig. 3:**
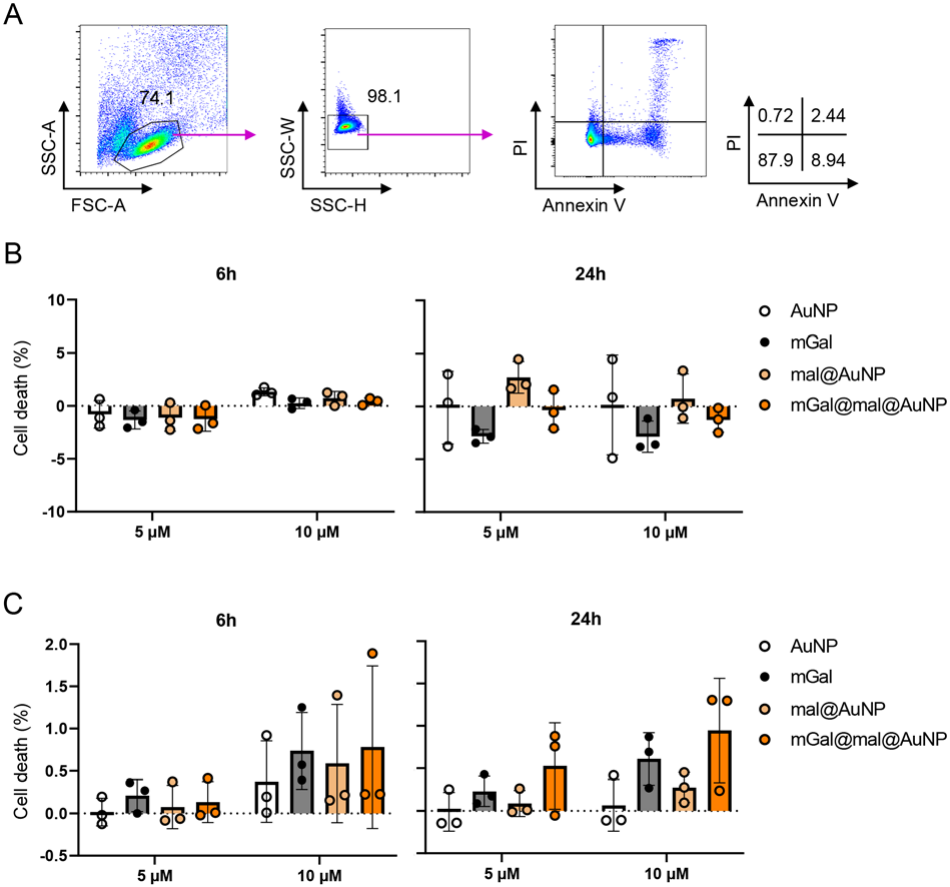
Apoptosis effect of galectin–nanoparticle conjugates (mGal@mal@AuNP) on PBMCs. (A) Gating strategy used to identify early (annexin V^+^PI^−^) and late (annexin V^+^PI^+^) apoptotic cells. Numbers represent the percentage of mother populations. (B and C) Cell death (%) of (B) early and (C) late apoptotic cells, calculated as follows: cell death = (% of annexin V^+^PI^+^ cells with stimulus minus the percent annexin V^+^PI^+^ cells without stimulus)/(annexin V^−^PI^−^ cells without stimulus) × 100. Anti-CD3 mAb-activated PBMCs were treated with 5 μM and 10 μM of each compound. Apoptosis was measured at 6 hours (left) and 24 hours (right) after stimulation. Each symbol represents one donor. Data in Fig. 3B and C are shown as mean ± SD. Abbreviation: AuNP, gold nanoparticle; mGal, galectin protein; mal@AuNP, maleimide–nanoparticle conjugate; mGal@mal@AuNP: galectin–maleimide–nanoparticle conjugate.

Given the known selective proapoptotic activity of Gal-1 towards activated splenocytes,^5,31,32^ we evaluated the capacity of mGal@mal@AuNP to promote apoptosis of activated human T cells. Thus, we exposed anti-CD3 mAb-activated human peripheral blood mononuclear cells (PBMCs) to all the nanoparticles at two different concentrations, 5 μM and 10 μM (concentration of mGal), and used a flow-cytometry-based assay to assess the pro-apoptotic effect of Gal-1-bearing NPs (mGal@mal@AuNP). The gating strategy used to analyse the flow cytometry data is shown on Fig. 3A. For each experiment, the unconjugated gold nanoparticles (AuNP), the maleimide conjugated AuNP (mal@AuNP) and the unconjugated galectin (mGal) were used as controls. We considered the effect of the NPs at two different time points, 6 h and 24 h. Interestingly, Gal-1-bearing nanoparticles were not effective in inducing early apoptotic cells (Fig. 3B). However, induction of late apoptotic death was much more pronounced and was dose-dependent (Fig. 3C). Thus, Gal-1-conjugated nanoparticles did not show loss of activity. Overall, at both concentrations, the induction of apoptosis was similar or slightly higher than that observed with the unconjugated protein.

### 2.4 Gal-1-conjugated nanoparticles induce apoptosis of Jurkat T cells

**Fig. 4:**
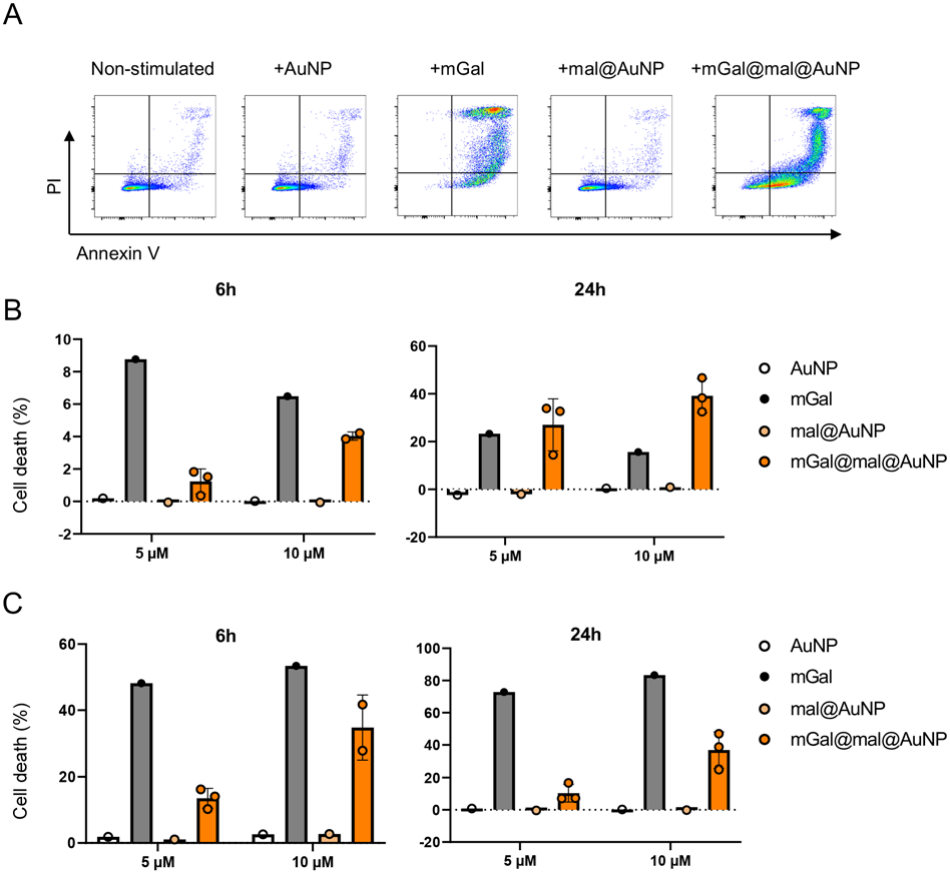
Pro-apoptotic effect of galectin–nanoparticle conjugates (mGal@mal@AuNP) on Jurkat T cells. (A) Represented flow cytometry dot plots. Cells were treated with 10 μM of each compound and measured 24 h post stimulation. Cells were pre-gated for size and singlets. (B and C) Cell death (%) of (B) early and (C) late apoptotic cells were calculated as follows: cell death = (% of annexin V^+^PI^+^ cells with stimulus minus the percent annexin V^+^PI^+^ cells without stimulus)/(annexin V^−^PI^−^ cells without stimulus) × 100. Jurkat T cells (1 × 10^5^ cells) were treated with 5 μM and 10 μM of each compound. Apoptosis was measured at 6 h (left) and 24 h (right) after stimulation. Each symbol represents compounds from different patches. Data in Fig. 4B and C are shown as mean ± SD. Abbreviation: AuNP, gold nanoparticle; mGal, galectin protein; mal@AuNP, maleimide–nanoparticle conjugate; mGal@mal@AuNP: galectin–maleimide–nanoparticle conjugate.

Jurkat cells are a widely used, immortalized human T lymphocyte cell line derived from T-cell acute lymphoblastic leukemia, serving as a valuable tool for studying T-cell biology in health and disease.^33^ Thus, the pro-apoptotic effect of mGal@mal@AuNP was also evaluated in the Jurkat T cell line, using two different concentrations (5 μM and 10 μM) and two time points (6 h and 24 h), using a similar flow-cytometry-based assay. Fig. 4A depicts flow cytometry dot plots after stimulation. As in Fig. 3, unconjugated gold nanoparticles (AuNP and mal@AuNP) and the unconjugated galectin (mGal) were used as controls. The unconjugated protein had strong pro-apoptotic effects at all time-periods and concentrations analysed, both early and late apoptotic cells, while the unconjugated gold nanoparticles had no effect on cell death. After 24 h, the Gal-1-conjugated nanoaparticles induced 25–40% cell death both at 5 μM and 10 μM. In each case, it outperformed with regards to the unconjugated proteins. The unconjugated protein at both concentrations was very effective in inducing late apoptotic cell death at both 6 h (∼50% death) and 24 h (∼70% death) after treatment. In comparison, mGal@mal@AuNP could induce ∼10% cell death at 5 μM and ∼40% death at 10 μM.

### 2.5 Gal-1-bearing nanoparticles induce secretion of IL-10, an immunoregulatory cytokine

**Fig. 5:**
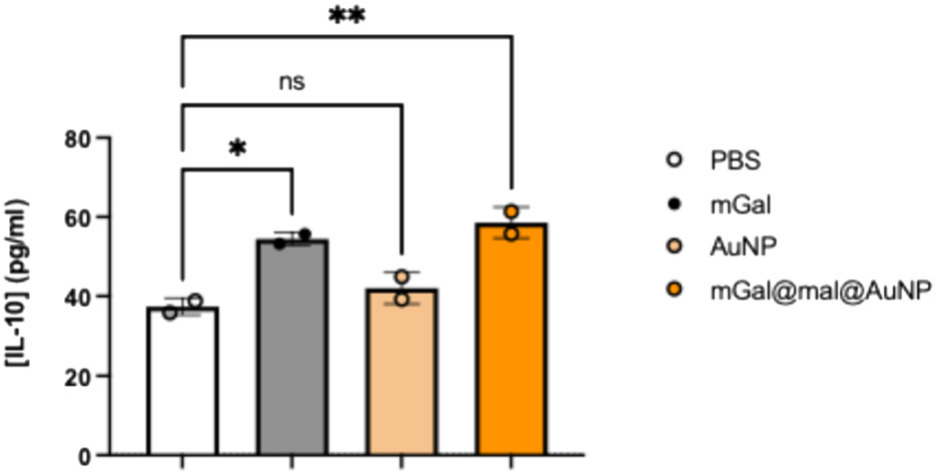
IL-10 secretion of THP-1 cells treated with PBS, recombinant Gal-1 or the synthesized NPs, determined by ELISA. Abbreviation: AuNP, gold nanoparticle; mGal, galectin protein; mal@AuNP, maleimide–nanoparticle conjugate; mGal@mal@AuNP: galectin–maleimide–nanoparticle conjugate.

Added to its ability to induce T-cell apoptosis at high concentrations, lower concentrations of Gal-1 induce IL-10 secretion from T cells and monocytes/macrophages.^6,34^ Thus, we evaluated secretion of IL-10 from THP-1, a widely used human monocytic leukemia cell line, following treatment with recombinant Gal-1, or the nanoparticles (Fig. 5). Notably, we observed higher IL-10 secretion for THP-1 cells treated with mGal@mal@AuNP, than with PBS control or with naked AuNPs.

## 3. Discussion

Nanoparticles have been designed for use in biosensors, fluorescent tags at the molecular scale, imaging agents, targeted molecular delivery systems, and other valuable biological tools, featuring chemically modifiable surfaces that allow for the attachment of different ligands, including lectins.^35–38^ Our study provides a proof of concept of the capacity of Gal-1-conjugated gold nanoparticles as a potential immunotherapeutic strategy for treatment of T cell-mediated autoimmune and chronic inflammatory disorders. The successful synthesis, functionalization, and characterization of these conjugates set the stage for further *in vivo* evaluations and potential clinical applications.

Apoptotic assays revealed that mGal@mal@AuNP exhibit enhanced early apoptotic activity compared to unconjugated Gal-1, inducing 25–40% of early apoptosis at 24 h. This suggests that multivalent Gal-1 presentation on the nanoparticle surface may potentiate apoptotic signalling. However, in late apoptosis, unconjugated Gal-1 remained more effective, inducing up to 70% cell death at 24 hours, while mGal@mal@AuNP showed a dose-dependent response, reaching ∼40% at 10 μM. The differential apoptotic effects between early and late stages apoptotic effects may be attributed to nanoparticle uptake pharmacodynamics, Gal-1 release, or apoptotic pathway activation.

Furthermore, cytokine release assays showed that Gal-1-bearing nanoparticles (mGal@mal@AuNP) enhance Il-10 secretion from monocytic THP-1 cells, even more than recombinant Gal-1, demonstrating the immunosuppressive potential of the synthesized nanoparticles.

These findings highlight the potential of mGal@mal@AuNP as an immunomodulatory agent. Future studies will be critical to elucidate the mechanistic basis of these effects and refine nanoparticle design to maximize therapeutic efficacy. *In vivo* validation in models of autoimmune disease, including rheumatoid arthritis, experimental autoimmune encephalomyelitis and inflammatory bowel diseases^5,15,39^ will be essential to confirm the therapeutic clinical relevance and safety of this therapeutic immunomodulatory approach.

## 4. Conclusions

In sum, we describe a novel approach involving multivalent presentation of Gal-1 on ultra-small nanoparticles, which may have potential applications of treatment for autoimmune diseases and chronic inflammatory disorders. A simple three step synthetic strategy to multivalently present galectins on glycosylated gold nanoparticles was developed. Importantly, after conjugation, Gal-1 retains or enhances its biological activity compared to unconjugated Gal-1.

## 5. Material and methods

### 5.1 Production and purification of recombinant human galectin-1

Gal-1 was produced and purified as outlined previously.^40^ In brief, *E. coli* BL21 (DE3) cells were transformed with expression plasmids constructed using pET expression systems (Novagen) and production of recombinant Gal-1 (mGal) was induced by the addition of 1 mM isopropyl-β-d-thiogalactoside (IPTG). Soluble fractions were obtained for subsequent purification by affinity chromatography on a lactosyl-sepharose column (Sigma-Aldrich). mGal was eluted with PBS buffer supplemented with 100 mM lactose and the eluted fractions were then dialyzed against PBS to remove bound lactose. LPS content of the purified samples was depleted using Detoxigel endotoxin-removing columns (Thermo-Fischer Scientific). Finally, Gal-1 samples were sterilized by passage through a 0.22 μm syringe filtered and stored at −80 °C until further use.

### 5.2 Synthesis of glucose-stabilized gold nanoparticles (AuNP)

The glucose-stabilized nanoparticles were synthesized using a previously published protocol.^29^ In a representative synthesis, sodium 1-thio-β-d-glucose (Glc-SNa) (500 μL, 41.2 mM) was added to HAuCl_4_ (6.25 mL, 2.89 mM) at room temperature. Instantly, a change in colour from yellow to brown was observed which indicated the formation of the gold nanoclusters. This suspension was vortexed for five minutes until the colour turned dark brown. The solution was then transferred to a falcon tube with a filter and centrifuged at 3000*g* for 30 minutes. This procedure was repeated three times and finally the residue was diluted with water. UV visible spectra of the resultant solution were measured and the number of AuNP was calculated using a previously reported protocol.^27^

### 5.3 Synthesis of maleimide-conjugated gold nanoparticles (mal@AuNP)

AuNPs (1 mL, 200 nmol) were diluted with 1 mL of water. To this solution, 4 μmol of 1-ethyl-3-(3-dimethylaminopropyl)carbodiimide (EDC, Alfa-Aesar), 4 μmol of *N*-hydroxysulfosuccinimide (sulfo-NHS, Aldrich) and 1 μL of *N*,*N*-diisopropylethylamine (DIPEA) was added and sonicated for five minutes. Then, to this solution, 4 μmol of 1-(2-aminoethyl) maleimide hydrochloride (Sigma) was added and the mixture was sonicated for 2 h at room temperature. Then, the contents were dialysed (Spectra/Por® dialysis tubing, diameter 4.6 mm, MWCO 6–8 kDa) in 2 L water overnight. The contents of the dialysis tube was then passed through a 0.45 μm (FP 30/0.45 Whatman, GE) filter and stored at 2–8 °C until further usage.

### 5.4 Synthesis of Gal-1 conjugated gold nanoparticles (mGal@mal@AuNP)

Mal@AuNPs (90 μL, 7 nmol mL^−1^) were diluted with PBS (4 mL). To this solution, 800 μg of Gal-1 (100 μL from 8 mg mL^−1^ solution) protein was added and stirred overnight. Then, the contents were dialysed (Spectra/Por^®^ dialysis tubing, diameter 4.6 mm, MWCO 6–8 kDa) in 1.5 L water overnight. The content of the dialysis was stored at 2–8 °C until further use.

### 5.5 Infrared spectroscopy

All measurements were performed using a Perkin Elmer Spectrum 100 FT-IR spectrometer. Aqueous dispersions of glyconanoparticles were first lyophilized and then resuspended in 20 μL methanol. The methanolic suspensions (5 μL) were dropped on the probe to dry before applying pressure gauge to record the infrared spectrum. The transmittance spectra furnished in the main text were base-line corrected and slightly smoothed for presentation.

### 5.6 Transmission electron microscopy (TEM)

TEM measurements were performed on a Zeiss EM 912 Omega. The samples were prepared by gently dropping the samples (20 μL) onto grids and subsequent solvent evaporation in a dust protected atmosphere.

### 5.7 AFM characterization and analysis

Samples were prepared on freshly cleaved Mica and dried at room temperature. AFM images were acquired using a commercial AFM system (JPK NanoWizard 3 and 4). Measurements were performed in AC Mode with SNL-10 probes (Bruker) at 25 °C, 35–40% RH. AFM images were collected with 1024 ×1024 pixels per frame. Each AFM tip was characterized prior to usage. Analyses of AFM images were performed with the JPK Data Processing software. Note that for the height analyses of the AFM images, the baseline height was levelled against the flat base plane of the substrate. All AFM images were only subjected to the primary first order flattening correction to remove sample tilt so that potential artifacts induced by other image processing steps were avoided as much as possible.

### 5.8 Cell death assays

Jurkat T cells (1 × 10^6^ cells per ml), purchased at ATCC, were incubated with or without 5 or 10 μM WT recombinant human Gal-1 (mGal), mal@NPs or mGal@mal@NPs in RPMI medium supplemented with 1 mM β-mercaptoethanol. PBMCs from healthy donors were isolated by density gradient centrifugation (Ficoll-Paque), and T cells were activated by incubation with 1 μg ml^−1^ of agonist anti-CD3 and anti-CD28 antibodies. After 3 days, cells were washed and incubated in fresh RPMI supplemented with 5% FCS and antibiotics in the presence or absence of 5 or 10 μM WT Gal-1, mal@NPs or mGal@mal@NPs in RPMI medium supplemented with 1 mM β-mercaptoethanol. Apoptotic cells were identified by staining with fluorescein isothiocyanate-conjugated annexin V (BD Biosciences) and propidium iodide (PI). Cell death was determined as: (% of annexin V^+^PI^+^ cells with stimulus minus the percent annexin V^+^PI^+^ cells without stimulus)/(annexin V^−^PI^−^ cells without stimulus) × 100.

### 5.9 Cytokine secretion assay

The THP-1 cell line was obtained from ATCC (THP-1 – TIB-202) and cultured following the suggested conditions. Cells were incubated with AuNPs, (mGal), or mGal@mal@AuNP in RPMI medium during 48 h at 37 °C. After incubation, cultured supernatants were collected and IL-10 secretion was analyzed by ELISA following the manufacturer's instructions (R&D DuoSet human IL-10 ELISA).

## Author contributions

C. G. and S. D. L. designed the project with support from P. H. S. and G. A. R. C. G. performed the synthesis and characterization of the nanoparticles with assistance from M. S. L. Y. and A. J. C. conducted the biological experiments with support from and S. D. L. C. G. analysed the data and wrote the manuscript with help from S. D. L. and A. J. C. P. H. S., G. A. R., edited the manuscript, provided funding, resources, and overall supervision of the project. All authors reviewed and approved the final manuscript.

## Conflicts of interest

There are no conflicts to declare.

## Data Availability

All data supporting the findings of this study are included in the main manuscript. Raw data files are available from the corresponding author upon reasonable request.
